# Identification of a new probiotic strain, *Lactiplantibacillus plantarum* VHProbi^®^ V38, and its use as an oral health agent

**DOI:** 10.3389/fmicb.2022.1000309

**Published:** 2022-12-12

**Authors:** Jingyan Zhang, Zhi Duan

**Affiliations:** Nutrition & Health Technology Center, Qingdao Vland Biotech Group Co., Ltd., Qingdao, China

**Keywords:** *Lactiplantibacillus plantarum* VHProbi^®^ V38, oral probiotics, caries, adhesion, biofilm, periodontal pathogenic bacteria

## Abstract

**Introduction:**

Probiotics can be used to treat oral diseases such as dental caries, gingivitis, periodontitis, and halitosis.

**Methods:**

This study screened for strains capable of inhibiting *Streptococcus mutans*,one of the primary pathogenic bacteria responsible for dental caries by agar diffusion in different samples. Strain identification was performed by 16S rDNA sequencing and the API 50CH system. The potential functions of the strains in terms of oral health properties were also tested by agglutination assays, growth inhibition assays, adhesion assays, biofilm removal assays and inhibition of adhesion in human primary gingival epithelial (HPGE) cells assays.

**Results:**

This study identified a probiotic strain from fermented cabbages that has a strong inhibitory effect on *Streptococcus mutans*. The API 50CH system and 16S rDNA sequencing verified that this was a new strain and it was given the name, *Lactiplantibacillus plantarum* VHProbi^®^V38. Agglutination, growth inhibition and adhesion, and biofilm removal tests indicated that *L. plantarum* VHProbi^®^ V38 inhibited and reduced *S. mutans*. This probiotic was shown to have a broad antibacterial spectrum, simultaneously inhibiting the growth of periodontal pathogenic bacteria such as *Porphyromonas gingivalis*, *Aggregatibacter actinomycetemcomitans*, and *Fusobacterium nucleatum*. After 2 hours of co-cultivation with these pathogens, *L. plantarum* VHProbi^®^ V38 was able to significantly reduce pathogens adhesion on human primary gingival epithelial (HPGE) cells.

**Discussion:**

These findings suggest that *L. plantarum* VHProbi^®^ V38 could potentially prevent and treat periodontal diseases caused by these pathogenic bacteria. *L. plantarum* VHProbi^®^ V38 also adheres strongly to HPGE cells and thus has potential as an oral probiotic. This study describes new methods that can be used to aid the screening and identification of oral probiotics.

## Introduction

Bacteria, archaea, fungi, protozoa, and viruses can all be found in the oral cavity. While each has a particular role, they can also interact strongly with each other and the host in both health and disease (Sampaio-Maia et al., 2016). Various risk factors, including host susceptibility, poor oral hygiene, or dietary habits, can modify the oral microbiota, altering the natural balance between commensal and pathogenic microorganisms (Esteban-Fernández et al., 2018; Fan et al., 2018). These changes can result in a predominance of opportunistic pathogens in the oral cavity that lead to bacterial infections such as pharyngitis, halitosis, caries, periodontitis, gingivitis, and other oral diseases (Amer et al., 2017; Gao et al., 2018). Dental caries, primarily caused by *Streptococcus mutans,* is a common biofilm-dependent oral disease in humans, which manifests as a progressive demineralization of calcareous tissues caused by the complicated interactions between acid-generating bacteria and fermentable carbohydrates (Duque et al., 2011; Wen et al., 2011; Bal et al., 2019). *Streptococcus mutans* is an acidogenic bacterium that grows in plaque and releases lactic, formic, butyric, propionic, and other organic acids while metabolizing carbohydrates (Wang et al., 2013). The organic acids demineralize tooth surfaces and initiate dental caries (ten Cate 2006). Periodontal disease, one of the main causes of tooth loss in adults, occurs in periodontal tissue. The most common pathogenic bacteria involved include *Porphyromonas gingivalis*, *Clostridium nucleatus*, and *Prevotella intermedia* (Mayanagi et al., 2009).

Probiotics are nonpathogenic microorganisms that can confer a positive health benefit on the host when consumed in adequate amounts (FAO/WHO, 2006). Traditionally, probiotics have been used to promote the health of the gastrointestinal tract (Connolly et al., 2010; Roberfroid et al., 2010; Willem et al., 2010; Hemarajata and Versalovic, 2013). However, in recent years interest in the use of probiotics for non-gut applications such as skin health (Bateni et al., 2013) and protection against dental caries (Maitra et al., 2013) have also emerged. Probiotics are primarily used in oral health applications to control cariogenic streptococci which colonize the mouth (Acharya, 2016) and manage diseases such as dental caries, periodontitis, halitosis, and candidiasis (Flichy-Fernández et al., 2010; Pradeep et al., 2014).

Recent studies indicate that probiotics play a positive role in oral health. *L. rhamnosus* GG, *L. plantarum*, and *L. reuteri* inhibit biofilm formation by *S. mutans* (Söderling et al., 2011; Amez et al., 2017), while *L. casei*, *L. plantarum* ST-III, and *L. paracasei* LPC27 reduce cell growth and the biofilm formation of *S. mutans* (Lin et al., 2017). The antibacterial components produced by *Lactobacillus* sp. include bacteriocin or bacteriocin-like substances, lactic acid, and hydrogen peroxide (Shanker and Federle, 2017). *Lactobacillus* can also antagonize the growth of periodontal pathogens such as *Actinomycetes aggregator*, *Prevotella intermedia*, *Fusobacterium nucleatum* and *Porphyromonas gingivalis* (Sookkhee et al., 2001; Kang et al., 2005; Koll-Klais et al., 2005).

The current study describes a new strain, *L. plantarum* VHProbi® V38, that was isolated from fermented cabbages and tested in several oral health-related assays. This novel strain was found to inhibit the growth of pathogenic bacteria, reduce the biofilm produced by pathogenic bacteria and colonize human mouth epithelial cells, potentially aiding the establishment of a new microecological balance in the oral environment.

## Materials and methods

### Bacterial strains and growth conditions

The pathogenic bacteria used in this study contained *Streptococcus mutans* ATCC 25175, *Streptococcus mutans* CCTCC AB 99010, *Streptococcus mutans* BNCC 700610, *Porphyromonas gingivalis* BNCC 353909, *Aggregatibacter actinomycetemcomitans* BNCC 336945, and *Fusobacterium nucleatum* BNCC 336949. The human primary gingival epithelial (HPGE) cells were purchased from iCell Company (Shanghai, China). Lactic acid bacteria (LAB) strains were grown on Man, Rogosa and Sharpe (MRS) plates for 48 h in anaerobic conditions at 37°C. *Streptococcus mutans* were grown on brain-heart infusion (BHI) plates for 24 h in aerobic conditions at 37°C. *Porphyromonas gingivalis, A. actinomycetemcomitans,* and *F. nucleatum* were grown on blood agar plates (Columbia Blood Agar Base), supplemented with 5% sheep blood for 48–72 h in anaerobic conditions at 37°C.

### Strain enrichment and screening

All samples (10 g) were weighed, diluted with 10 times the weight of normal saline, and slapped with a homogenizer for 5 min. The samples were serially diluted in tenfold steps. A portion of each sample (100 μl) was cultured at 10^−1^, 10^−2^ and 10^−3^ dilutions on MRS plates for 48 h in anaerobic conditions at 37°C. After colonies grew on the plates, those of different shapes were selected for gram staining and observed under a microscope. Rod-shaped and Gram-positive strains were identified as potential probiotics.

### Bacterial identification

The isolates were identified with a standard biochemical test using the API CH50 system (Biomerieux, Marcy, l’Etoile, France) and 16S rDNA sequence analysis. Polymerase chain reaction (PCR) was used to amplify 16S rDNA with the universal bacterial primers, 27F [5’-AGAGTTTGATCCTGGCTCAG] and 1492R [5’-GGTTACCTTGTTACGACTT]. The PCR products were then purified with a Wizard PCR Preps DNA Purification System and sequenced with a Big Dye TM Terminator Cycle Sequencing Ready Reaction Kit and a model 310 automatic sequencer. The closest known relatives of the new isolates were identified by database sequence searches, and the sequences of closely related strains were retrieved from the GenBank libraries or Ribosomal Database Project databases. A phylogenetic tree was constructed using the NJ (Neighbor-Joining) method with MEGA 6.0 software, and the bacteria were identified.

### Preparation of bacterial lysates

LAB isolates were grown in MRS plates overnight at 37°C. Tubes (15 ml) of overnight culture were placed in an ice bath and the cells were lysed by sonication for 20 min and then incubated for 60 min at 80°C. The lysates were tested for sterility on MRS plates.

### Bacteriostatic activity testing

Bacteriostatic activity was assessed using the agar diffusion method (Wang et al., 2017; Mao et al., 2020). *Streptococcus mutans* ATCC 25175, *S. mutans* CCTCC AB 99010, and *S. mutans* BNCC 700610 were initially used as indicator strains. The three strains were precultivated in the BHI broth both separately and mixed together as indicator strains. All experiments were performed in duplicate. Growth inhibitory (GI) activity was calculated by measuring the diameter of the inhibition zone.

Strains with an inhibitory effect on *S. mutans* were used to test the growth inhibition against a broader spectrum of oral indicator bacteria. These included *P. gingivalis*, *A. actinomycetemcomitans,* and *F. nucleatum*, pathogens associated with gingivitis, periodontitis, and halitosis, respectively (Koll-Klais et al., 2005).

### Coaggregation testing

Coaggregation was studied using a visual assay (Cisar et al., 1979). LAB isolates were incubated in MRS broth overnight at 37°C. Three strains of *S. mutans* were separately incubated in BHI broth overnight at 37°C. *Porphyromonas gingivalis*, *A. actinomycetemcomitans,* and *F. nucleatum* were separately incubated in ATCC2722 broth (Supplemented Tryptic Soy broth containing 0.1 mg/100 ml Vitamin K1 and 0.5 mg/100 ml Hematin chloride) for 24–48 h at 37°C.

Cultures were harvested when the optical density at 600 nm reached 1.0–1.5. The bacteria were collected by centrifugation at 8000 rpm for 10 min, washed twice, and resuspended with an equal volume of buffer (pH 7.0 with 0.025 M potassium phosphate containing 0.025 M NaCl). The initial OD600 absorbance was adjusted to 0.5–0.6. LAB suspension (300 μl) was added to a 24-well plate and 300 μl of the pathogenic bacteria suspension was added as a reaction sample. The same amount of LAB suspension and buffer mixture was used as a control. Two parallel suspensions were created for each control and sample. The *S. mutans* suspension included a mixture of the three strains. The 24-well plates were shaken on a rotary micro shaker for 120 min at room temperature and agglutination was observed.

### Inhibition of the growth of *Streptococcus mutans*

The *L. plantarum* VHProbi® V38 lysate was prepared according to section 2.4. Then it was filtered through a 0.22 μm microporous membrane, and the supernatant was prepared. The three strains of *S. mutans* were incubated separately for 24 h at 0.1% inoculum (v/v). The amount of bacteria was approximately 2.0 × 10^8^ CFU/ml. The three strains were then mixed in equal volumes. Bacteria were collected by centrifugation, and then was washed twice with 7.0 phosphoric acid buffer (PBS), resuspended in an equal volume of PBS and then diluted 5 times with PBS, and used as an inoculation solution. BHI broth medium (50 μl) with a 4x concentration was added to each well of the 96-well plates, 20 μl (or 50 μl) original supernatant prepared and 10 μl inoculation solution were then added, respectively, and the volume was replenished to 200 μl with sterile water. The supernatant concentration (v/v) in the 200 μl volume was 10% (20 μl) and 25% (50 μl) respectively. Liquid paraffin (50 μl) was added to each well for sealing. In the blank control group, the buffer solution was used instead of the supernatant. Each array created four parallel groups. A growth curve was generated to assess the effect of the supernatant on the growth of *S. mutans*.

### Antagonistic adhesion testing

The inhibitory effect of *Lactobacillus* sp. on *S. mutans* adhesion was performed as previously reported (Söderling et al., 2011). In brief, *Lactobacillus strains* and the mixture of the three *S. mutans* strains were washed twice with PBS. The initial OD600 absorbance was adjusted to 0.5–0.6. *S. mutans* and *Lactobacillus* strains were mixed at equal ratios. A upper layer of solution (500 μl) was added to the 24-well plate with chamber slides and cultured at 37°C for 2 h. The supernatant was discarded, and the unadhered bacteria in the wells were washed twice with PBS. Methanol (0.5 ml) was added to the wells, fixed for 10 min, and discarded. The wells were then stained with 300 μl Giemsa stain for 10 min, the stain was discarded, and the wells were rinsed with PBS. The chamber slides were removed and the number of *S. mutans* was observed using a bright-field microscope.

### Biofilm elimination testing

Biofilm assays were performed in polystyrene 24-well (flat-bottom) plates using the method by Khan et al. with some modifications (Khan et al., 2010). The overnight *S. mutans* cultures were transferred to a 24-well plate with chamber slides and incubated for 24 h to form a biofilm at 37°C under aerobic conditions. The plate was then washed twice with PBS. A portion of the tested samples (600 μl), including cell suspensions, fermented broth, or the lysate, was inoculated into the wells. The cell suspension was prepared according to the method in section 2.6. The lysate was prepared according to section 2.4. MRS broth alone was used as the control. After inoculation, all plates were incubated at 37°C for 24 h to measure the ability of the samples to remove biofilms. The culture medium was then decanted, and the plates were gently washed three times with 600 μl sterilized PBS to remove planktonic and loosely bound cells.

The biofilm removal rate was determined by counting the number of bacteria on the chamber slides. The chamber slides were taken out and placed in sterile homogeneous bags. The slides then received ultrasonic cleaning for 10 min to diffuse the thallus into the buffer solution. After a series of dilutions, 100 μl of the samples were cultured into the light salivary *Streptococcus* culture medium containing 200 U/l bacitracin to determine the concentration of *S. mutans*. The following formula was used to calculate the removal rate of biofilm: removal rate (%) = (1-amount in the experimental group/amount in the control group) *100.

### Adhesion testing on HPGE cells

The adhesion test described by Scaletsky et al. (1984) was performed with some modifications. Human primary gingival epithelial (HPGE) cells were cultured in 5% CO_2_ at 37°C in RPMI-1640 Media containing 10% (v/v) fetal bovine serum and 1% (v/v) penicillin–streptomycin solution. The adhesion test was performed in a 24-well chamber slide system. HPGE cells were seeded (2.5 × 10^5^ cells well^−1^) and incubated in 5% CO_2_ at 37°C for 18 h and briefly washed twice with the RPMI-1640 Media. The LAB isolates were then washed twice with PBS and resuspended with an equal volume of the medium. The OD600 absorbance was adjusted to 0.4–0.5. The wells were inoculated with 500 μl of the bacteria suspension and the plates were incubated at 37°C in 5% CO_2_ for 120 min to allow attachment. The cells were then washed three times with PBS, fixed with methanol, Giemsa stained for 5 min, washed with PBS, and air dried. Bacterial adhesion to each epithelial cell was observed using a bright-field microscope. Fifty cells were observed and counted under the microscope, and LAB isolates on the visible cell surface were quantified. The mean and standard deviation of the adhesion index was calculated using the formula: adhesion index = number of adhesion bacteria/number of cells × 100%.

### Antagonistic adhesion testing of HPGE cells

The inhibition of pathogen adhesion by *Lactobacillus* strains was performed using the Esteban-Fernández method with modifications (Esteban-Fernández et al., 2018). Adhesion tests were performed using a 24-well chamber slide system. The HPGE cells were seeded (2.5 × 10^5^ cells well^−1^) and incubated at 37°C in 5% CO_2_ for 18 h. The HPGE cells were briefly washed twice with the medium.

The fresh LAB and pathogen solutions (*P. gingivalis*, *A. actinomycetemcomitans*, or *F. nucleatum*) were washed twice with PBS and suspended with the 1,640 culture containing 10% fetal bovine serum in the same volume. The OD600 absorbance was adjusted to 0.4–0.5. The LAB and pathogenic bacteria suspension were mixed at a ratio of 1:1, and 500 μl of the mixture was added to the prepared 24-well chamber slide system. HPGE cells incubated with *P. gingivali*, *A. actinomycetemcomitans,* and *F. nucleatum* alone were used as controls. The plates were incubated in at 37°C in 5% CO_2_ for 120 min. The cells were then washed three times with PBS, fixed with methanol, Giemsa stained for 5 min, washed with PBS, and air dried. Bacterial adhesion on each epithelial cell was observed using a bright-field microscope. Fifty cells were observed and counted under a microscope, and pathogenic bacteria on the visible cell surface were calculated. The mean and standard deviation of the adhesion index was calculated using the formula: adhesion index = number of adhesion bacteria/number of cells × 100%.

## Results and discussion

### Screening of oral probiotics

LAB strains (*n* = 237) were screened from raw materials from different sources including fermented cabbages, yogurt, soy juice, and the oral cavity. Of these, 24 strains that inhibited the growth of *S. mutans* using agar diffusion methods were screened. The V38 strain screened from fermented cabbages was able to strongly prevent the growth of *S. mutans* with an inhibition zone of 2.75 ± 0.35 cm. The strain lysate also had an inhibitory effect on *S. mutans*, with an inhibition zone of 2.50 ± 0.15 cm.

The antibacterial properties of *L. plantarum* VHProbi® V38 were further investigated. The bacterial liquid and lysate also had an inhibitory effect on *P. gingivalis*, *A. actinomycetemcomitans,* and *F. nucleatum* (Table 1). This experiment showed that the strain had a broad antibacterial spectrum on oral pathogens. The fermentation broth had a slightly better bacteriostatic effect than the lysate, and the effect on *S. mutans* was the strongest.

**Table 1:** Inhibition zone on other pathogenic bacteria (cm).

Samples	Indicator organism			
	*S. mutans*	*P. gingivalis*	*A. actinomycetemcomitans*	*F. nucleatum*
The fermentation broth	2.75 ± 0.35	1.55 ± 0.07	1.20 ± 0.01	1.05 ± 0.05
The lysate	2.50 ± 0.15	1.30 ± 0.06	1.10 ± 0.05	1.00 ± 0.03
The lysate (pH7.0)	1.60 ± 0.15	0.80 ± 0.01	0.80 ± 0.01	0.80 ± 0.01

When the pH of the lysate was adjusted to 7.0, it was found that the lysate still had inhibition against *S. mutans*, while it was less effective against *P. gingivalis*, *A. actinomycetemcomitans* and *F. nucleatum*. It shows that in addition to the inhibitory effect of acid on pathogenic bacteria, other inhibitory substances are present in metabolic substances.

### Bacterial identification

The isolated V38 strain was characterized using biochemical tests and found to ferment ribose, galactose, glucose, fructose, mannose, mannitol, sorbitol, α-methyl-D-mannoside, N-acetylglucosamine, amygdaline, arbulin, aesculin, salicin, cellobiose, maltose, lactose, melibiose, sucrose, trehalose, inulin, raffinose, gentiobiose, turanose, gluconate and 2-ketogluconate. The strain was defective in fermenting glycerol, erythritol, D-arabinose, L-arabinose, D-xylose, L-xylose, adonitol, β-methyl-D-xyloside, sorbose, rhamnose, dulcitol, inositol, α-methyl-D-glucoside, melezitose, amidon, glycogen, xylitol, D-lyxose, D-tagatose, D-fucose, L-fucose, D-arabitol and 5-ketogluconate.

**Figure 1:**
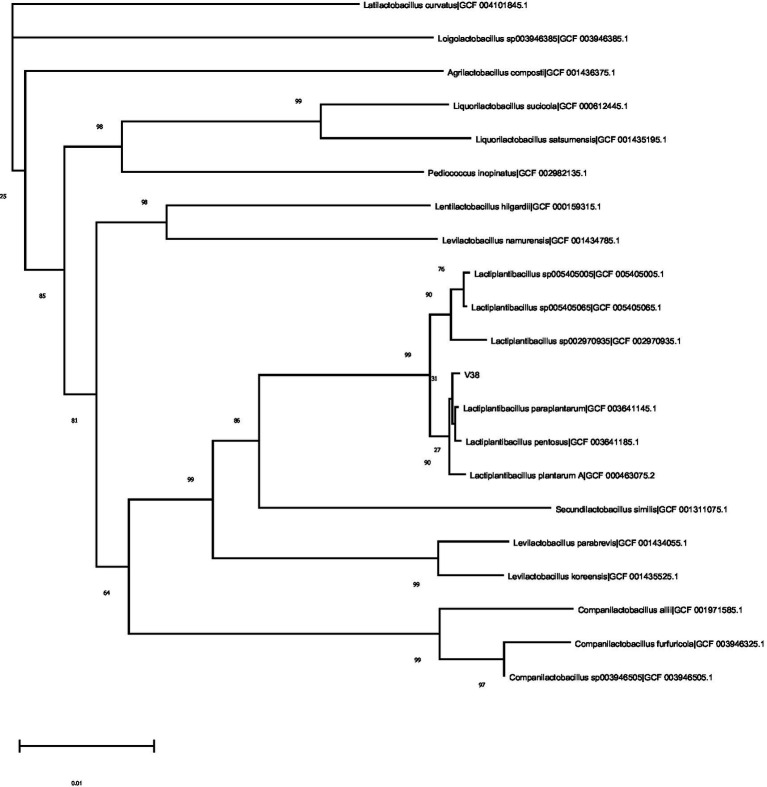
Phylogenetic tree based on 16S rDNA sequences of *Lactiplantibacillus plantarum* and related taxa.

A tree depicting the phylogenetic position of the V38 strain is shown in Figure 1. A nearly complete 16S rDNA sequence^1^ identified the V38 strain as *Lactiplantibacillus plantarum* and showed that it shared marked similarity with the species reference strain. Based on the test results, the V38 strain was identified as a new strain and was named *Lactiplantibacillus plantarum* VHProbi® V38.

### Coaggregation testing

Coaggregation is a highly specific process involving interactions between bacterial surface molecules that act as adhesins and complementary receptors such as proteins and carbohydrates (Palmer et al., 2003; Kang et al., 2005). Probiotics can prevent dental caries by agglutinating with pathogenic microorganisms to prevent their colonization and adhesion in the mouth. *Lactobacillus* can combine with pathogenic bacteria to form visible flocculent precipitation under certain conditions.

**Figure 2:**
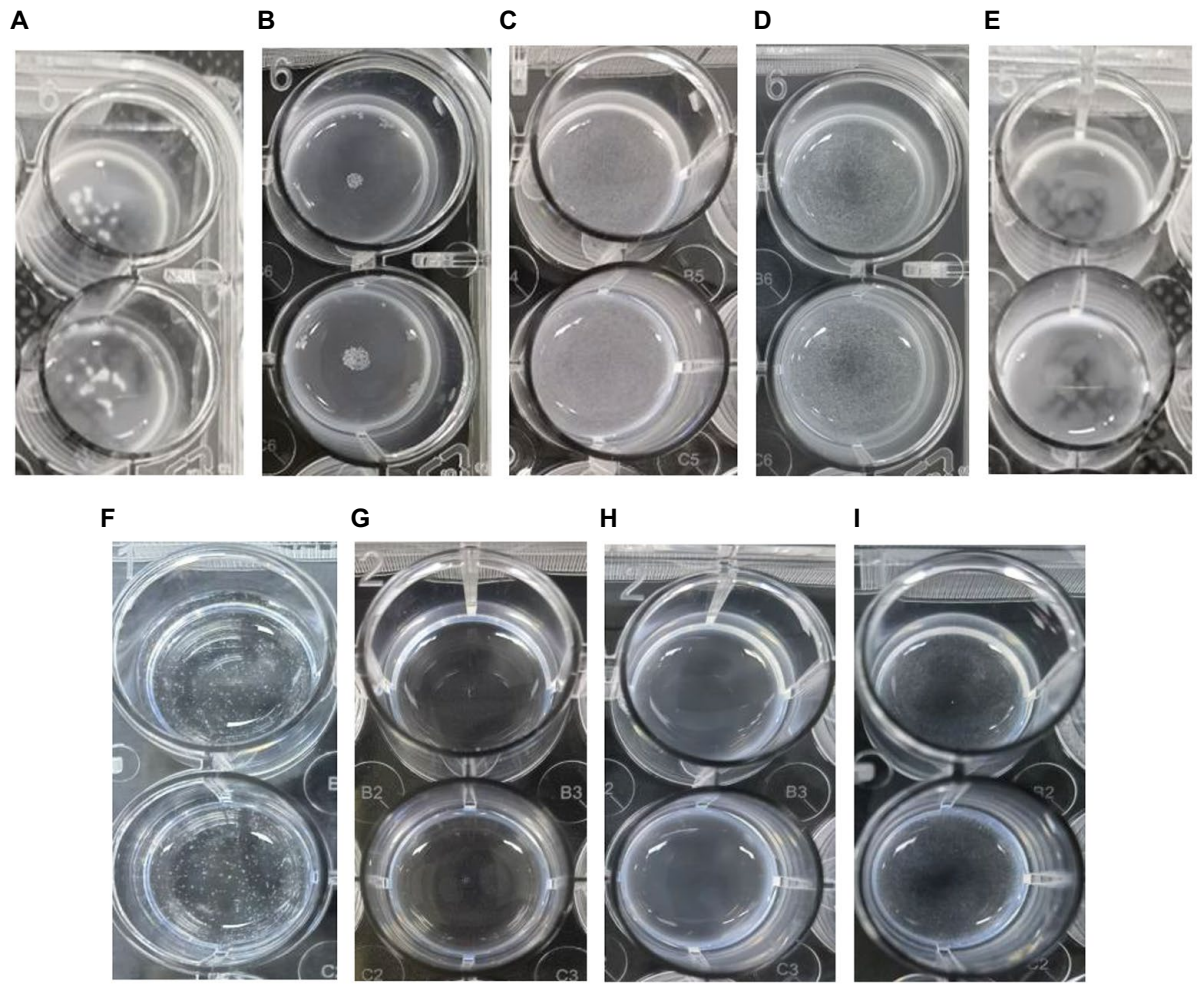
Coaggregation of *L. plantarum* VHProbi® V38 with four pathogenic bacteria. **(A)** *L. plantarum* VHProbi® V38 + *S. mutans*; **(B)** *L. plantarum* VHProbi® V38 *+ A. actinomycetemcomitans*; **(C)** *L. plantarum* VHProbi® V38 + *P. gingivalis*; **(D)** *L. plantarum* VHProbi® V38 + *F. nucleatum*; **(E)** *L. plantarum* VHProbi® V38 control; **(F)** *S. mutans* control; **(G)** *A. actinomycetemcomitans* control; **(H)** *P. gingivalis* control; **(I)** *F. nucleatum* control.

Within 2 h, *L. plantarum* VHProbi® V38 had created large, aggregated points with *S. mutans* and *A. actinomycetemcomitans* (Figures 2A,B). The V38 strain had smaller, dense, aggregated points with *P. gingivalis* and *F. nucleatum*, indicating that it bound less strongly with these organisms (Figures 2C,D). *L. plantarum* control didn’t have any agglutination points (Figure 2E). *S. mutans* control had some smaller agglutination points (Figure 2F). *A. actinomycetemcomitans* and *P. gingivalis* control did not have any agglutination point (Figures 2G,H). *F. nucleatum* control had had some small agglutination points (Figure 2I). The levels of aggregation differ for each strain and are dependent on time (Twetman et al., 2009), indicating that not all *Lactobacillus* strains can aggregate with harmful bacteria. Aggregation assays may be a useful complement to screening probiotic candidates with possible anti-caries properties. Indeed, the ability of *L. plantarum* VHProbi® V38 to coaggregate with harmful bacteria is an important indicator of its potential value as an oral probiotic.

### Effect of the V38 strain supernatent on *Streptococcus mutans* growth

**Figure 3:**
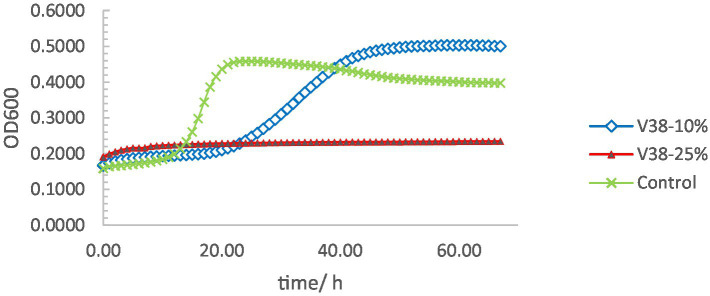
Growth curves of *S. mutans* under different supernatant concentrations.

The supernatant of *L. plantarum* VHProbi® V38 contained fermentation metabolites and soluble components of the thallus and was able to significantly inhibit the growth of *S. mutans* (Figure 3). In the control group, *S. mutans* began to grow rapidly after 10 h and reached a growth plateau after 20 h. However, in the experimental group containing a 10% concentration of *L. plantarum* VHProbi® V38 supernatant, *S. mutans* had almost no growth within 20 h. *S. mutans* began to grow with additional time, however, indicating that the 10% supernatant could not inhibit its growth after 20 h. In the experimental group supplemented with 25% supernatant, *S. mutans* had very limited growth within 70 h, indicating that a high concentration of supernatant can more effectively inhibit this pathogen.

### Anti–adhesion and biofilm removal testing

**Figure 4:**
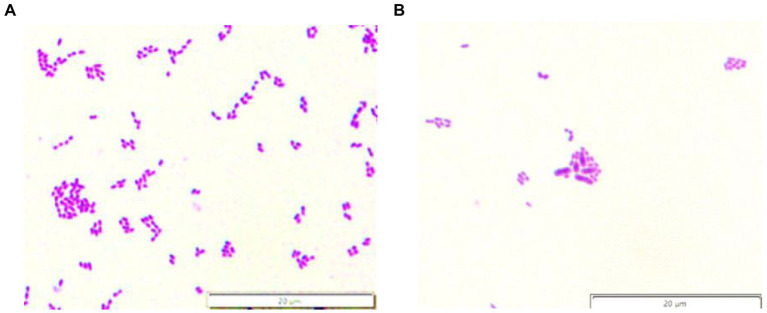
*S. mutans* adherence to chamber slides. **(A)** *S. mutans* control; **(B)** the experimental.

*Streptococcus mutans* is recognized as a primary pathogenic bacteria of caries (Beighton, 2005), and its colonization and adhesion in the oral cavity is the main reason for the formation of dental caries. Thus, oral probiotics must adhere well to harmful oral bacteria, inhibit their growth, and effectively diminish oral disease (Comelli et al., 2002; Kang et al., 2005). The adhesion test is used to determine if *S. mutans* adhesion on the teeth can be reduced after co-culture with probiotics. While *S. mutans* covered the control chamber slides (Figure 4A), the number was significantly reduced in the experimental slides (Figure 4B). *Lactiplantibacillus plantarum* VHProbi® V38 was able to effectively inhibit the adhesion of *S. mutans* to the chamber slides, indicating that it could potentially prevent pathogenic bacteria from adhering to the teeth and inhibit dental caries.

*Streptococcus mutans* forms plaque biofilms through surface protein binding to sites on acquired membranes on the tooth surface. The inhibition and removal of dental plaque biofilm are important for preventing caries (Lee and Kim, 2014). *In vitro* studies suggest that probiotics can prevent the adhesion of *S. mutans* strains to saliva-coated hydroxyapatite and interfere with biofilm formation (Haukioja et al., 2008; Söderling et al., 2011; Schwendicke et al., 2017). *Lactiplantibacillus plantarum* VHProbi® V38 was able to inhibit *S. mutans* biofilm formation. While the control chamber slides had 277,000 CFU of *S. mutans,* the fermented broth, cell suspension, and lysate groups had 0 CFU, 130,000 CFU, and 0 CFU, respectively. The removal rate of biofilm in the fermented broth, cell suspension, and lysate groups was 100, 53.07, and 100%, respectively. While the fermented broth and lysate were able to completely remove the biofilm and potentially prevent and treat *S. mutans*-induced dental caries, the cell suspension was able to remove about 53% of the biofilm. These findings illustrate that *L. plantarum* VHProbi® V38 is likely able to remove *S. mutans* biofilm either directly or through the secretion of antibacterial substances.

### Adhesion testing of HPGE cells

Adherence is an important prerequisite for the colonization of probiotics in the oral cavity, providing a competitive advantage in this ecosystem (Lamont and Jenkinson, 2000). Oral epithelial cell monolayer testing is a method used to identify beneficial bacteria and has also been used to measure the attachment of beneficial bacteria to the oral epithelium. According to an earlier study, if the number of attached bacteria per HPGE cell is ≥ 1.5, the attachment capacity is considered to be very strong and if the number of attached bacteria is 1.5–1, the adhesion capacity is considered to be strong. If the number of attached bacteria is 1–0.5, the ability to adhere is moderate while if the number is < 0.5, the ability to adhere is considered weak (Jin et al., 2000). *Lactiplantibacillus plantarum* VHProbi® V38 had very strong adhesion to HPGE cells. After 2 h of cultivation, about nine bacteria adhered to each HPGE cell, and the adhesion index was 8.67 ± 1.59. The strong adhesion capacity of these bacteria indicate that they can remain in the oral cavity for a longer time and likely play a more effective probiotic role.

### Adhesion antagonism of *Porphyromonas gingivalis*, *Aggregatibacter actinomycetemcomitans* and *Fusobacterium nucleatum*

**Figure 5:**
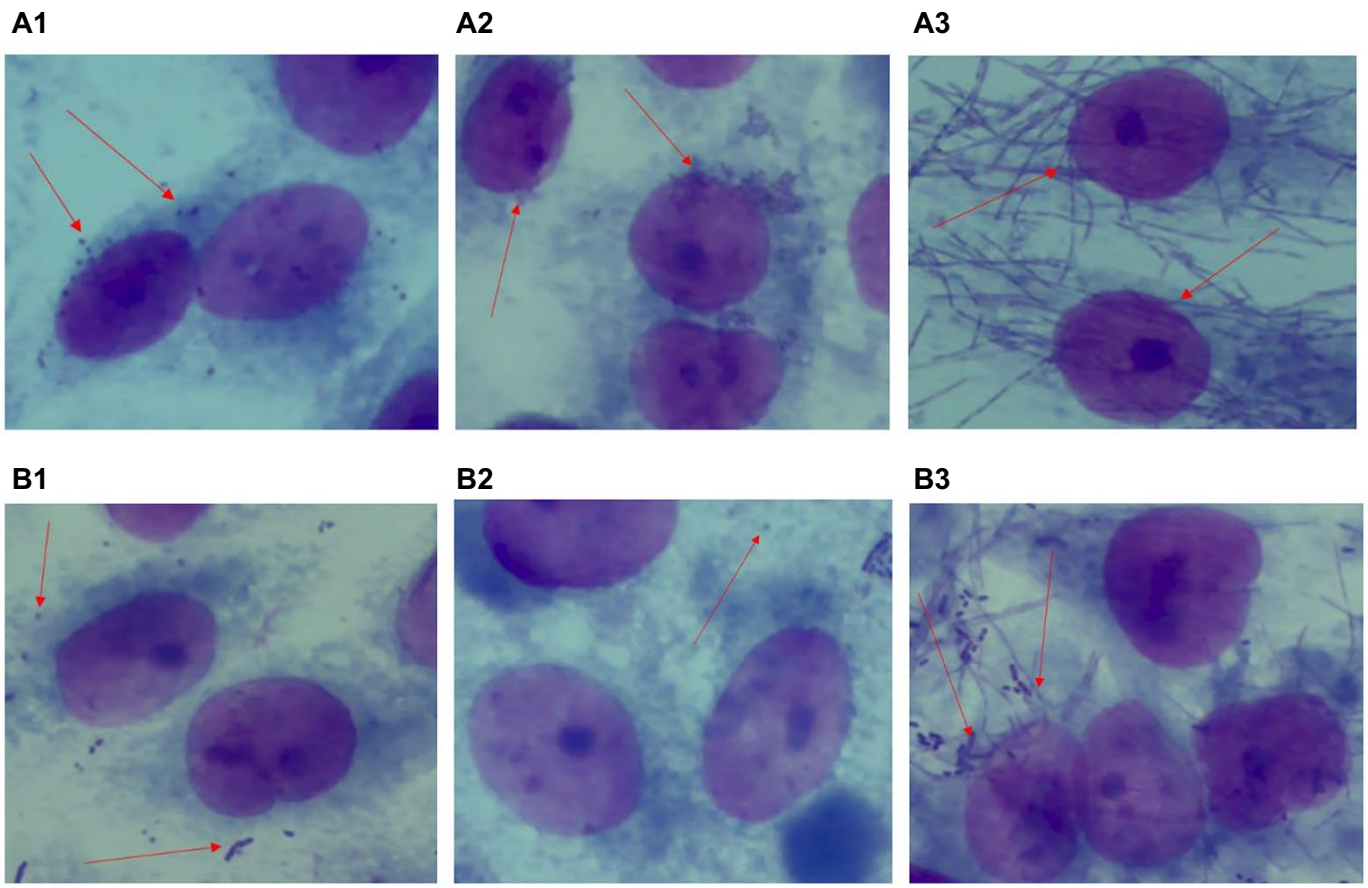
Antagonistic adhesion of *L. plantarum* VHProbi^®^ V38 to *P. gingivalis*, *A. actinomycetemcomitans* and *F. nucleatum* **(A1)** control *P. gingivalis*; **(A2)** control *A. actinomycetemcomitans*; **(A3)** control *F. nucleatum*; **(B1)** *L. plantarum* VHProbi^®^ V38 + *P. gingivalis*; **(B2)** *L. plantarum* VHProbi^®^ V38 + *A. actinomycetemcomitans*; **(B3)** *L. plantarum* VHProbi^®^ V38 + *F. nucleatum*.

*Lactiplantibacillus plantarum* VHProbi® V38 had a significant inhibitory effect on the adhesion of *P. gingivalis*, *A. actinomycetemcomitans*, and *F. nucleatum*, common periodontal pathogens in the oral cavity. Fifty cells in the microscope’s field of vision were selected and the number of pathogenic bacteria on the cell surface was quantified. *P. gingivalis*, *A. actinomycetemcomitans* and *F. nucleatum* all had a strong adhesion force on HPGE cells (Figures 5A1-A3). The adhesion index was determined by calculating the number of pathogenic bacteria on the cell surface and was shown to be 20.8 ± 7.13 for *P. gingivalis*, 36 ± 6.56 for *A. actinomycetemcomitans*, and 32.8 ± 2.54 for *F. nucleatum*. After a 2-h co-culture of *L. plantarum* VHProbi® V38 with each pathogen, the adhesion index decreased to 1.92 ± 0.67 for *P. gingivalis* (Figure 5B1), 1.1 ± 0.82 for *A. actinomycetemcomitans* (Figure 5B2), and 3.7 ± 0.69 for *F. nucleatum* (Figure 5B3). *L. plantarum* VHProbi® V38 greatly reduced the adhesion of *P. gingivalis*, *A. actinomycetemcomitans*, and *F. nucleatum* to HPGE cells. The adhesion indexes of all control and experimental groups were significantly different (*p* < 0.05).

### Discussion

While several studies have explored the potential benefit of probiotics on oral health (Campus et al., 2014; Lin and Pan, 2014), there is no standardized or comprehensive protocol for screening novel oral probiotics *in vitro*. Effective probiotics should have one or more of the following properties: growth inhibition of pathogenic bacteria (Simark-Mattsson et al., 2007; Teanpaisan et al., 2011), coaggregation with pathogenic bacteria (Re et al., 2000; Lang et al., 2010), inhibition of *S. mutans* biofilm formation (Zhang et al., 2020), interference with *S. mutans* colonization of teeth (Khan et al., 2010) and competitive exclusion of pathogen adhesion to cells (Wang et al., 2014).

The current study screened a strain of *L. plantarum* that inhibited the growth of *S. mutans* using enrichment and agar diffusion methods. The strain was identified as novel using the API 50 CH system and 16S rDNA sequence analysis and named *L. plantarum* VHProbi® V38. The V38 strain was found to inhibit the growth of *S. mutans* on agar plates and in liquid culture. *S. mutans* growth in liquid culture was inhibited within 70 h when a 25% supernatant of the V38 strain was included. The new strain could also form visible agglutination points with *S. mutans*, *P. gingivalis*, *A. actinomycetemcomitans,* and *F. nucleatum*. Coaggregation interactions help to establish and maintain biofilms and are related to adhesion (Kolenbrander, 2000; Re et al., 2000; Lang et al., 2010). These *in vitro* experiments demonstrated that *L. plantarum* VHProbi® V38 could potentially be used to prevent and treat dental caries caused by *S. mutans. L. plantarum* VHProbi® V38 could potentially colonize the oral cavity, and agglutinate and adhere to *S. mutans* to quickly remove a portion of the biofilm. When the pH of the lysate was adjusted to 7.0, it was found that the lysate still had inhibition against *S. mutans*. The *L. plantarum* may secrete bacteriocin to inhibit the growth of *S. mutans* and produce other substances that bind to the surface of the pathogen cell membrane. Additional studies will be required to better identify the substances involved in this process.

In addition to inhibiting *S. mutans*, *L. plantarum* VHProbi® V38 can also inhibit periodontal pathogens such as *P. gingivalis*, *A. actinomycetemcomitans* and *F. nucleatum*, using a broad antibacterial spectrum. After a 2-h co-culture with these periodontal pathogens, *L. plantarum* VHProbi® V38 was able to reduce pathogen adhesion on HPGE cells. These findings indicate the strain’s antibacterial ability against oral pathogens. *L. plantarum* VHProbi® V38 has strong adhesion to HPGE cells, indicating that it could be used to colonize the oral cavity and serve as an oral probiotic. Since lysates can also inhibit pathogenic bacteria, preventing *S. mutans* adhesion and removing the biofilm, the lysate of the V38 strain also has the potential to prevent and treat oral diseases.

The current study not only screened a lactic acid bacterium that could potentially prevent and treat oral caries and periodontal disease but also described new methods for the study of potential oral probiotics. Further clinical research is needed to verify the efficacy of *L. plantarum* VHProbi® V38 as a novel probiotic.

## Data availability statement

The datasets presented in this study can be found in online repositories. The names of the repository/repositories and accession number(s) can be found at: OP077205.1, https://www.ncbi.nlm.nih.gov/nuccore/OP077205.

## Author contributions

JZ: conceptualization, methodology, data curation, and writing. ZD: methodology, investigation, supervision, and writing-reviewing and editing. All authors contributed to the article and approved the submitted version.

## Conflict of interest

JZ and ZD were employed by Qingdao Vland Biotech Group Co., Ltd.

## Publisher’s note

All claims expressed in this article are solely those of the authors and do not necessarily represent those of their affiliated organizations, or those of the publisher, the editors and the reviewers. Any product that may be evaluated in this article, or claim that may be made by its manufacturer, is not guaranteed or endorsed by the publisher.

## Footnotes

^1^ https://www.ncbi.nlm.nih.gov/nuccore/OP077205
